# Production of secondary metabolites under challenging environments: understanding functions and mechanisms of signalling molecules

**DOI:** 10.3389/fpls.2025.1569014

**Published:** 2025-08-11

**Authors:** Sana, Tariq Aftab, M. Naeem, Prakash Kumar Jha, P. V. Vara Prasad

**Affiliations:** ^1^ Plant Physiology Section, Department of Botany, Aligarh Muslim University, Aligarh, India; ^2^ Department of Plant and Soil Sciences, Mississippi State University, Starkville, MS, United States; ^3^ Feed the Future Innovation Lab for Collaborative Research on Sustainable Intensification, Kansas State University, Manhattan, KS, United States; ^4^ Department of Agronomy, Kansas State University, Manhattan, KS, United States

**Keywords:** abiotic stress, antioxidants, cross-talk, secondary metabolites, signalling molecules, metabolic regulation

## Abstract

Plants are sessile organisms confronted by various abiotic stresses, including drought, salinity, heavy metals (HMs), and high/low temperatures throughout their growth cycles. In response to stress conditions, plants activate a cascade of metabolites and signalling molecules and networks. These intricate networks of signalling molecules like nitric oxide (NO), hydrogen sulfide (H_2_S), methyl jasmonate (MeJA), hydrogen peroxide (H_2_O_2_), ethylene (ETH), melatonin (MT), and calcium (Ca^2^ **^+^**), play a crucial role in enhancing the production of secondary metabolites (SMs) in plants. In plants, SMs are characterized by four diverse groups’ terpenes, phenolics, alkaloids, and glucosinolates. Various environmental factors and plant developmental stages influence the production of SMs. The production and regulation of terpenes, phenolics, alkaloids, and glucosinolates in response to signalling molecules under stressed conditions provide valuable insights into stress tolerance. These insights are crucial for developing agricultural practices that improve crop resilience. They are essential for plants to cope with oxidative stress by providing defence mechanisms for improved adaptation, tolerance, and resilience strategies. Conversely, the crosstalk among the signalling molecules paves the way for new research avenues of plant stress management. This review emphasizes the essential role of SMs in plants and how the signalling molecules regulate their production under stress conditions. It also provides valuable insights into the mechanisms that facilitate plant adaptation and stress resilience.

## 1 Introduction

Plants constantly face a variety of environmental stress factors, including biotic (e.g., pathogens, insects) and abiotic (e.g., drought, salinity, and temperature). To cope with these stresses, plants initiate various defence mechanisms by synthesizing secondary metabolites (SMs) or compounds. Secondary metabolites are bioactive compounds in plants that are essential for biological activities (Erb and Kliebenstein, 2020; Jan et al., 2021) and play important roles in plant survival, adaptation, and resilience. Secondary molecules can alleviate the detrimental symptoms in plants caused by a stressful environment and improve their efficiency and metabolic activity (Kessler and Kalske, 2018; Salam et al., 2023). Under adverse circumstances, plants produce more than 100,000 SMs using biosynthetic pathways (Meena et al., 2017). Secondary metabolites can be categorized into 4 major groups: terpenes, phenolics, alkaloids (nitrogen-containing), and glucosinolates (sulfur-containing). Monoterpenes, inclusive of menthol, linalool, camphor, α-pinene, and β-pinene, have antimicrobial and antioxidant activities (Reshi et al., 2023). Volatile compounds like terpenoids provide stress tolerance to plants against oxidative and thermal stressors (Bai et al., 2019). Compared to normal conditions, more phenolic and flavonoid compounds are biosynthesized by plants growing in stressful environments. Noticeably, flavonoids and anthocyanin compounds (which exhibit antioxidant capacity) reduce ROS (reactive oxygen species) and associated oxidative damage (Sharma et al., 2019). Various internal and external factors, including stress and signalling molecules, significantly influence these metabolite productions. Unlike traditional signalling molecules, such as hormones, gasotransmitters (GTs) diffuse rapidly through plant tissues and interact with various cellular targets to regulate biochemical and physiological pathways. They play a significant role in modulating complex stress signalling pathways and metabolic processes (Sana et al., 2025).

Among the myriads of signalling molecules involved in regulating plant secondary products, nitric oxide (NO), hydrogen sulfide (H_2_S), methyl jasmonate (MeJA), hydrogen peroxide (H_2_O_2_), ethylene (ETH), melatonin (MT), and calcium (Ca^2+^) have emerged as key players. Nitric oxide is a signalling molecule involved in various physiological processes in plants, including growth and metabolic activities. Nitric oxide stimulates or inhibits specific enzymes and transcription factors, thereby influencing the biosynthetic pathways of SMs. Being a dynamic molecule, NO provides adaptation to plants under adverse environmental conditions (Zhou W. et al., 2021; Kumari R. et al., 2024). Hydrogen sulfide, a gaseous molecule similar to NO, has recently gained attention due to its pivotal role in plant physiology and reactivity to stress (Zhang et al., 2021; Saini et al., 2024). Hydrogen sulfide mitigates the adverse effects of abiotic stress by counteracting the accumulation of ROS, such as peroxides, hydroxyl radicals (OH^−^), superoxide radicals (O_2_^•–^), and singlet oxygen (O_2_). Reactive oxygen species disrupt nucleic acid structures and interfere with plant metabolic pathways (Amir et al., 2021). Many studies have been conducted on different plants under numerous stresses, showing that H_2_S application helps to cope with stress by enhancing bioactive compounds in plants (Lu et al., 2024; Shen et al., 2024). Similarly, jasmonic acid (JA) and its methyl ester derivative (MeJA) are known to produce broad categories of SMs, such as rosmarinic acid, terpenoids, plumbagin, and indole alkaloids (Almagro et al., 2014; Khan et al., 2024a). Notable increases in the expression of transcription factors and genes involved in forming SMs have been reported. For instance, WRKY (transcription factor engaged in biotic and abiotic stress responses) is the main factor that influences alkaloid production, such as taxol and artemisinin in *Taxus chinensis* and *Artemisia annua*, respectively (Subramaniyam et al., 2014). Besides, H_2_O_2_, ETH, MT, and Ca^2+^ also play integral roles in plant stress responses and SM production. They work individually or in a network of other molecules to enhance plant resilience. This process promotes metabolic adjustments and improves the accumulation of beneficial compounds. For instance, proline, carotenoids, glutathione, ascorbic acid, and phenolics are among the substances that are generated to control the harm that oxidative damage imparts. Ultimately, their action contributes to improved plant health and productivity in challenging environments (Chen H. et al., 2022; Zhang et al., 2021; Gupta et al., 2023). All these signalling molecules act as messengers, facilitating communication within plant tissues and activating specific pathways in response to environmental stimuli. This review discusses the multifaceted role and essentiality of signalling molecules in various abiotic stress conditions. Furthermore, it improves our understanding of the complex dynamic between signalling molecules and SMs to also improve the potency of these compounds in the agricultural sectors.

## 2 Secondary metabolites in plant’s defence and adaptation: an overview

**Figure 1:**
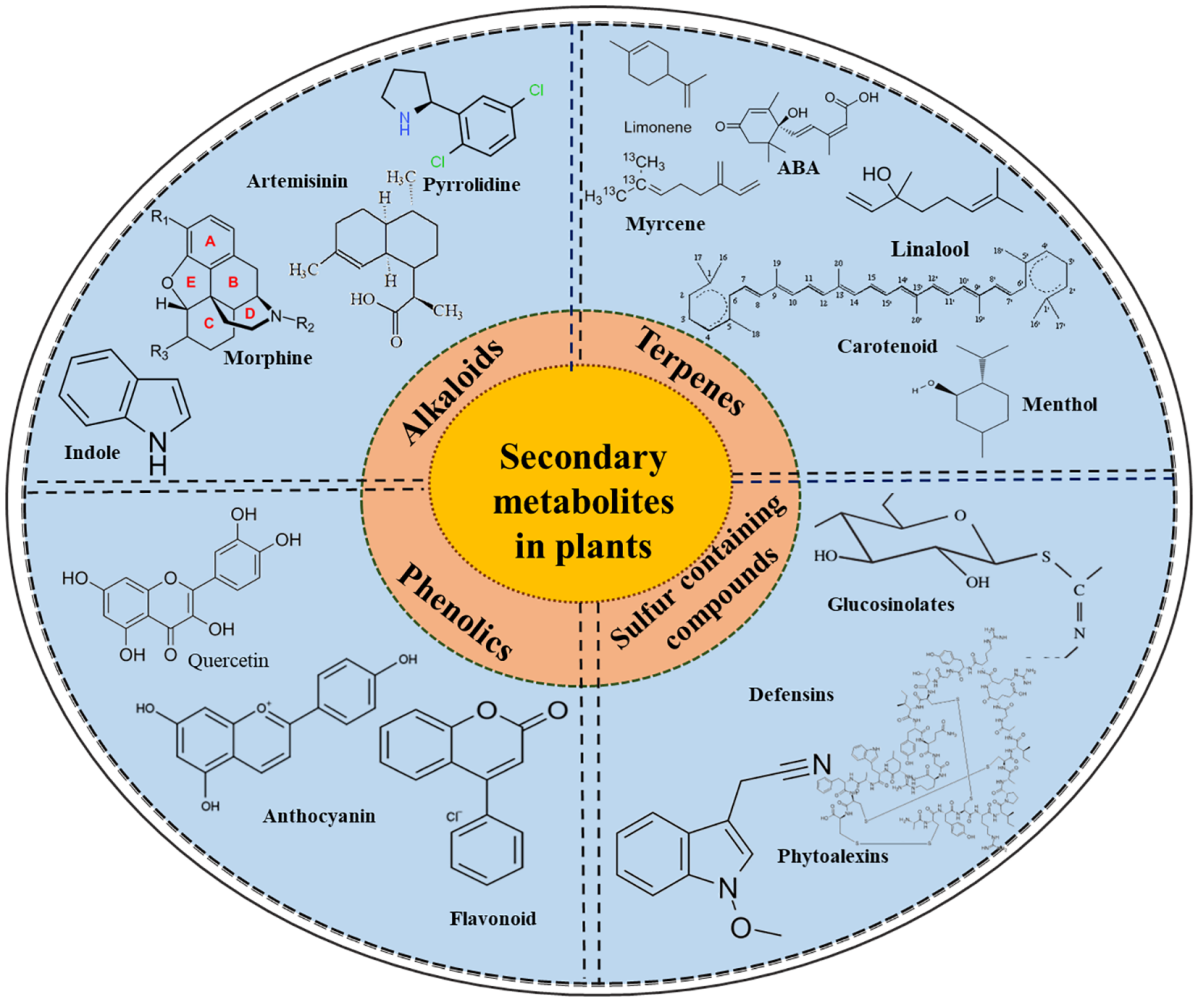
The schematic diagram shows the different classes of secondary metabolites, including terpenes, phenolics, alkaloids, and glucosinolates, along with some of their important members, which encompass diverse biological and physiological functions.

Primary and secondary metabolites are the two groups in which plant metabolites are distributed. Primary metabolites, such as proteins, lipids, and carbohydrates, directly influence plant growth and development. In contrast, SMs are small organic molecules originating from primary metabolites. Under specific circumstances, SMs serve particular functions, including resistance against pathogen and insect attacks and tolerance to abiotic stresses (Isah, 2019; Upadhyay et al., 2025). They contain a molecular mass of less than 3000 daltons and have widespread application in the agriculture sectors (Elshafie et al., 2023). In fact, plants have an assortment of molecular, cellular, and signalling crosstalk in stress response, which is triggered by the detection of certain abiotic stress factors that induce the generation of SMs. These metabolites are synthesized by multiple metabolic pathways and are implicated in the activation and reinforcement of defence mechanisms in plants. According to Patil (2020), these metabolites are classified into four broad categories: terpenoids (carbon and hydrogen compounds), phenolics (containing a benzene ring), alkaloids (nitrogen-containing compounds), and glucosinolates (sulfur-containing compounds), as shown in **Figure 1**.

### 2.1 Terpenoids

Terpenes or terpenoids are an enormous chemical group containing nearly 22,000 compounds. Isopentenyl pyrophosphate (IPP) and its isomer dimethylallyl pyrophosphate (DMAPP) bear five-carbon precursor units, which produce a basic ring of terpenoids. Plants have an active cytosolic mevalonic acid pathway (MVA) and plastidial 2-C-Methyl-D-erythritol-4-phosphate (MEP) pathways, which are both involved in the synthesis of IPP and DMAPP precursors (Bergman et al., 2024). DMAPP and IPP condense to form sesquiterpenes, sterols, and triterpenes. Meanwhile, the MEP pathway uses glyceraldehyde-3-phosphate, pyruvate, and 1-deoxy-D-xylulose 5-phosphate synthase (DXS) to produce DXP (Kumari M. et al., 2024). Terpenoids are distributed throughout the plants and play essential roles in plant physiology and ecology. They are also involved in environmental adaptation and stress tolerance (Boncan et al., 2020; Ninkuu et al., 2021). Furthermore, the terpenes are divided into monoterpenes (C_10_), sesquiterpenes (C_15_), diterpenes (C_20_), triterpenes (C_30_), tetraterpenes (C_40_), and polyterpenes with more than ten carbon units (C>10) (Naji et al., 2024). Also, terpenoids encompass carotenes, carotenoids, and xanthophylls, which function primarily as light-harvesting pigments and antioxidants. These pigments protect the photosystem by eliminating singlet oxygen production, scavenging ROS, and releasing surplus energy as heat through the xanthophyll cycle (Tiku, 2018; Khan et al., 2024a). Terpenoids act as membrane stabilizing agents, preventing ion leakage, and are used in natural pesticides and herbicides due to their repellent or toxic effects on pests (Khan et al., 2024a). Moreover, isoprene biosynthesis in plants is activated by high temperature, solar radiance, and water scarcity. Isoprene is proficient in quenching several ROS and NOS species and enhancing the thylakoid membrane constancy. Diterpenes and monoterpenes act as toxins or signalling molecules to deter pathogens. For instance, diterpenes help plants defend against microbes like *Magnaporthe oryzae*. In the case of rice, four main labdane-related diterpenoids momilactones A and B, phytocassanes A–F, oryzalexins A–F, and oryzalexin S. that act as phytoalexins (Zhan et al., 2020). The plant-parasitic nematode *Heterodera schachtii* was defended against by sesquiterpenoid nootkatone through the upregulation of defence genes controlling the SA (PR1, PR5, and NPR1) and JA pathways (JAZ10, CYP82C2, and LOX) (Habash et al., 2020).

### 2.2 Phenolics

Phenolic compounds, also known as phenols, are a diverse class of bioactive secondary compounds. The molecules have at least one aromatic ring containing one or more hydroxyl groups, ubiquitous in plants and essential for defending against pests, diseases, and abiotic stresses (Bano et al., 2023). The hydroxyl group helps counterbalance ROS like H_2_O_2_ and singlet oxygen, catalyses oxygenation reactions by forming metal complexes, and hinders the activity of certain oxidizing enzymes. An increased number of phenolic hydroxyl groups provides more reactive sites for oxidation, thereby strengthening the compound’s free radical neutralization capacity (Naikoo et al., 2019; Wang et al., 2021). Additionally, phenolic compounds differ in their ability to chelate metals, largely determined by their reduction potentials, with gallic acid at the top of the hierarchy and coumaric acid at the bottom. They also interact with membrane phospholipids and proteins. This interaction stabilizes cell membranes by reducing fluidity and preventing lipid peroxidation. They also facilitate numerous physiological and biochemical activities, including plant protection against biotic and abiotic stress (Al Mamari, 2021). Phenolic biosynthesis in plants is initiated by two major compounds: phosphoenolpyruvate (a component of glycolysis) and erythrose-4-phosphate (a component of the pentose phosphate pathway) via the shikimate pathway, which ultimately produces more complex and diverse phenolics compounds (Santos-Sánchez et al., 2019). Phenolic compounds act as antioxidants, scavenging ROS such as O_2_^•–^, H_2_O_2_, and OH. Moreover, phenolics can chelate (bind) with metal ions, preventing them from participating in ROS formation and increasing the activity of antioxidant-forming enzymes (Dias et al., 2021). Phenolics are divided into two groups: flavonoids and non-flavonoids. Two aromatic rings and a 15-C molecule comprise the molecular structure of flavonoids. Non-flavonoids contain complex structures and high molecular mass as compared to flavonoids. The primary function of phenolics is to provide colouration to various plant parts (Caleja et al., 2017). Flavonoids play diverse roles in plants, including antimicrobial agents, repellents, and UV protectants, as well as in the growth and development of various species (Mathesius, 2018). These are primarily sourced from vacuoles, which engage in ROS scavenging in peroxisomes, chloroplasts, and mitochondria. The interplay between phenolics and flavonoids majorly contributes to effective stress management in plants. Analogously, intense light, heat, nutrient, and sugar deficiencies promote anthocyanin accumulation in plants to prevail against adverse conditions. Some phenolic compounds help in synthesizing phytoalexins, which are antimicrobial substances produced in response to infection. The buildup of coumarins has been demonstrated to strengthen resistance to bacterial, viral, and fungal infections, which is especially noticeable in the defence against pathogens, such as oomycetes. Gallic acid pre-treatment in *Camellia sinensis* cv. Longjing 43 against *Ectropis obliqua* larvae stimulates the production of antifeedant compounds, like epigallocatechin-3-gallate, naringenin, and astragalin (Zhang X. et al., 2022). Likewise, purple corn pericarp extract enriches with polyphenols, which dramatically suppresses *Manduca sexta* growth (Tayal et al., 2020).

Together, phenolics and terpenoids provide a complementary, multi-layered defence strategy, integrating rapid biochemical responses with oxidative defences and enabling plants to resist stresses effectively. These two classes of secondary metabolites often function synergistically. Phenolics stabilize the cellular redox environment and limit pathogen spread (Shiade et al., 2024), and terpenoids act as early, front-line deterrents, either killing or repelling invaders or signalling for further defence responses.

### 2.3 Alkaloids

Nitrogen-containing compounds are produced from diverse aromatic amino acids in plants and can be categorized into alkaloids, cyanogenic glycosides, and non-protein amino acids. Based on their heterocyclic ring system and biosynthetic precursors, indole, purine, quinoline, isoquinoline, tropane, and imidazole alkaloids are classified into various classes (Roy, 2017). Non-protein amino acids act as precursors by providing the nitrogen atoms for alkaloid biosynthesis, which produces quinoline and quinazoline alkaloids. Synthesized alkaloids are stored in designated cellular compartments. In response to various environmental stress signals, these alkaloids are secreted from their storage organelles or specialized glands and transported to target tissues (Bhambhani et al., 2021). Alkaloids contain various antibacterial and antiviral activities and provide defence against a variety of microbes. Specifically, aromatic amino acids (phenylalanine, tyrosine, and tryptophan) serve as precursors for certain alkaloids (isoquinoline and indole alkaloids) and are recognized as antiherbivore compounds (Debnath et al., 2018). Besides, *Brassica juncea*, a cadmium (Cd) accumulator, shows high biomass production under Cd-contaminated soil and alkaloid accumulation, which counteract the stress effect (Tan et al., 2021). Alkaloids are the main SMs that accumulate in *Sophora alopecuroides*, associated with nitrogen metabolism. Experimental studies suggest that the upregulated unigenes in the nitrogen metabolism pathway may enhance alkaloid biosynthesis in *S. alopecuroides* under severe drought stress conditions (Huang et al., 2024). These substances also responded to mechanical stress by diminishing pathogen proliferation and inducing a hypersensitive response by programmed cell death (PCD) (Gogoi et al., 2024).

### 2.4 Sulfur-containing compounds

Glucosinolates, thiosulfate, allicin, derived from cysteine sulfoxides, reactive sulfur species (RSS) (H_2_S and sodium sulphate), and antimicrobial peptides (defensins and thionins) are examples of sulfur-containing metabolites that shield plants from pathogenic microbes (Künstler et al., 2020). These compounds have ecological and physiological significance for plants and are valued by humans for their flavours and potential health benefits, including cancer protection. Earlier workers reported that the abundance of glucosinolates occurs in the Brassicaceae family (Blažević et al., 2015). The biosynthesis of glucosinolates begins in the early stage with the help of aliphatic, aromatic, or indole amino acids through chain elongation and, lastly, is stored in vacuoles (Sharma et al., 2024). Noticeably, various sulfur-containing metabolites, including glutathione, phytoalexins, alliins, and defensins, participate in plant defence against various abiotic stress stimuli (Twaij and Hasan, 2022). Moreover, RSS, such as H_2_S, plays an influential role in alleviating the negative effects of abiotic stress in plants. For instance, in maize, H_2_S elevated the antioxidant enzyme and proline concentration, which suppressed abiotic stress consequences (Liu Z. et al., 2019). Similarly, phytoalexins (camalexin and resveratrol), low molecular weight compounds, are produced and accumulated through pathogenic infection to protect plants. One of the glucosinolates hydrolysis, 3-butene nitrile (3BN) elicits the defence in *A. thaliana*, by coordinating crosstalk with JA, SA, and NO signalling (Ting et al., 2020).

## 3 Signalling molecules in plants: an overview

**Figure 2:**
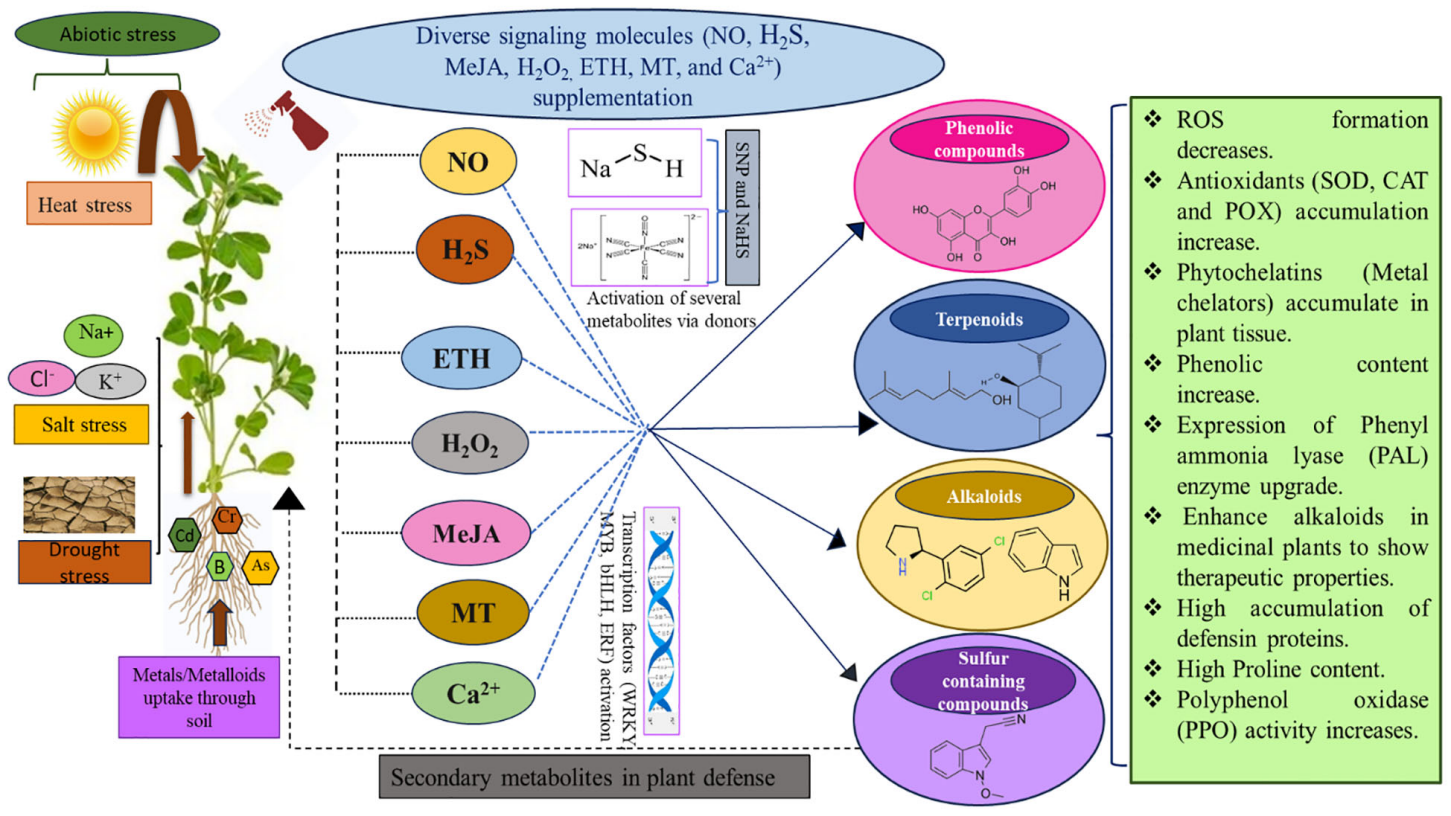
An overview of the role of signalling molecules (NO, H_2_S, ETH, H_2_O_2_, MeJA, MT, and Ca^+2^) under abiotic stress emphasizes their importance in strengthening plant resistance by regulating various pathways and enzymes regarding secondary metabolites. These metabolites trigger specific signalling cascades that help plants adjust to challenging environments. Fine-tuning protective mechanisms like antioxidant systems, osmotic balance, and stress-related gene expression contributes to improved plant tolerance and survival.

Signalling molecules, such as NO, H_2_S, MeJA, H_2_O_2_, ETH, MT, and Ca^2+^ in plants, facilitate communication within and between the cells under normal or stressful conditions and play decisive roles in physiological processes. They significantly impact the coordinated regulation of signalling networks and developmental processes under a wide range of challenging circumstances (Dikilitas et al., 2019). Optimal levels of signalling compounds trigger the activation of defence pathways, via the activation of enzymatic and non-enzymatic antioxidant systems, together with phenolics and other SM biosynthesis (Liang et al., 2018; Rao and Zheng, 2025). Signal transduction is a cascade of molecular events that convert extracellular physiological signals into intracellular responses. This response often leads to affecting the expression patterns of important genes and proteins. **Figure 2** highlights the role of various signalling compounds and their donors under numerous abiotic stresses, activating key genes that influence SM production and stimulating plant defence mechanisms.

### 3.1 Role of nitric oxide in SM production under stressful environment

Abiotic stress is a strong elicitor that recuperates SM production to impart signals in the plant, stimulating defence machinery and ensuring survival in harsh environments (Yeshi et al., 2022). Under stress conditions, plants usually do catabolic degradation of ROS into non-toxic compounds with the support of some enzymes, including superoxide dismutase (SOD), catalase (CAT), peroxidase (POX), and ascorbate peroxidase (APX). Plants have precise defence mechanisms to counteract the adverse situation and avoid the inimical consequences of stressful environments (Jan et al., 2021). As a compact free radical, NO positively contributes to plant development and growth, impacting every phase from germination to senescence (Rather et al., 2020). Nitric oxide production occurs in organelles including the chloroplast, mitochondria, peroxisome, plasma membrane, and apoplast, with the help of oxidative and reductive pathways (Nabi et al., 2019). NO effectively protects them from oxidative damage by triggering the activities of enzymes that control intracellular ROS (Hancock and Veal, 2021). Within cellular signalling, NO acts downstream of primary signals, such as Ca²^+^, JA, cADPR, abscisic acid, H_2_O_2_, MAPK cascades, and cGMP, serving as a secondary messenger. It is necessary to balance the concentration of NO when reacting to different physiological and environmental stresses. Earlier reports highlighted the importance of NO donors, such as sodium nitroprusside (SNP), S-nitro cysteine (CySNO), and S-nitrsoglutathione (GSNO) that are employed to improve the defence ability of plants (Rahim et al., 2022). Sodium nitroprusside (SNP), a NO donor, plays a fundamental role in plant processes and growth regulation and boosts alkaloid accumulation and antioxidant-related genes under stress conditions (Farzaei and Sayyari, 2024). Nitric oxide has been successfully employed as an elicitor to stimulate SM accumulation in plants. Nitric oxide under stressful conditions modulates non-enzymatic antioxidants, including ascorbic acid (AsA), glutathione (GSH), alkaloids, α-tocopherol, carotenoids, phenolics, flavonoids, and proline (Hasanuzzaman et al., 2020). For example, SNP applied on *Calendula officinalis* L. significantly enhanced the total flavonoids, phenolics, antioxidant activity, and essential oil yield (Zhang et al., 2012). Endogenously and exogenously produced NO and the application of NO donors regulate phenolics, flavonoids, and caffeic acid derivatives in several plant species (**Table 1**).

**Table 1:** Exploring the role of sodium nitroprusside (SNP) in managing secondary metabolite production in diverse plant species under abiotic stress.

Abiotic stress	Experimental plants	Concentration of SNP (NO donor)	Alteration in secondary metabolite production	References
Drought	*Melissa officinalis*	100 µM SNP	Leaf phenolic compound↑, total phenolic content↑, and PAL enzyme activity ↑.	(Safari et al., 2023)
	*Artemisia annua*	200 µM SNP	Alkaloids such as artemisinin ↑, Phenolic content ↑, and antioxidant activity↑.	(Wani et al., 2024)
	*Silybum marianum*	100 µM SNP	Amount of flavonolignans (Silybinin A and B) ↑, flavonoid (taxifolin)↑.	(Zangani et al., 2018)
	*Ocimum basilicum*	2 mM SNP	Terpenoids and acyclic terpenes, including Methyl chavicol↑, linalool↑, α-pinene↑, β- pinene↑, and myrcene↑.	(Esmaielpour et al., 2019)
Salt	*Crocus sativus*	10 µM SNP	Flavonoid content ↑, phenols accumulation ↑, Lycopene, and β-carotene↑.	(Babaei et al., 2021)
	*Salvia officinalis*	100 µM SNP	Total phenolics and total flavonoid content ↑, PAL activity ↑.	(Sheikhalipour et al., 2024)
	*Chenopodium quinoa*	25 µM SNP	PAL activity↑, saponin metabolite accumulation ↑, terpenoids such as ABA, and carotenoid production ↑.	(Jafari et al., 2024)
	*Centaurium erythraea*	250 µM SNP	PAL enzyme stimulation↑, resulting in a total phenolic amount in shoots ↑.	(Trifunović-Momčilov et al., 2023)
	*Triticum aestivum*	100 µM SNP	Total phenolic content ↑and oxidative burst↓.	(Ali et al., 2017)
Heavy metal	*Salvia officinalis*	200 µM SNP	Total phenolic content ↑, flavonoid content↑, and rosmarinic acid ↑ under Cu toxicity.	(Pirooz et al., 2022)
	*Solanum lycopersicum*	100 µM SNP	Phenolic components ↑, carotenoid content↑, under Cd stress, antioxidants (SOD, CAT, and POX) ↑, and ROS formation↓.	(Ahmad et al., 2018)
	*Raphanus sativus*	100 µM SNP	Phenolics ↑, flavonoids ↑, and anthocyanins ↑, metal chelation↑, ROS toxicity↓.	(Bhardwaj et al., 2023)

↑, Increase; ↓, Decrease.

#### 3.1.1 NO under drought stress

Drought stress is one of the severe threats to plants. Recent climate deviations have increased the frequency, intensity, and duration of stress, making it a global concern. Supplementation of NO produces diverse SMs and essential oil components. For example, 60 μM of SNP to *Origanum majorana* reduced lipid peroxidation and activated the PAL (phenyl ammonia lyase) enzyme, enhancing phenolics and a broad class of antioxidants (Liang et al., 2018; Farouk and Al-Huqail, 2020). In *Scrophularia striata*, the crosstalk of NO with H_2_O_2_ operates signal transduction pathways to induce PAL activity to enhance the accumulation of phenolic compounds under drought stress (Falahi et al., 2018). According to Chavoushi et al. (2020), the combined application of 25 μM SNP and 250 μM SA in safflower (*Carthamus tinctorius* L.) regulates the expression of some flavonoids (rutin, quercetin, luteolin, and apigenin) under drought conditions. They also stimulate the anthocyanins, phenols, and PAL activity. Like in *Radix saposhnikoviae*, SNP applied to fresh roots amplify the efficiency of acetyl-CoA carboxylase (ACC), PAL, and chalcone synthase (CHS) enzymes along with some therapeutic constituents, such as prim-*O*-glucosylcimifugin, cimifugin, and sec-*O*-glucosylhamaudol (Song X. W. et al., 2023). Under 60% field capacity, drought-affected *Brassica oleracea* displayed a significant increase in total phenolics content, chlorophyll, SOD, and POX activities. Foliar application of NO in the pre-sowing period raises the biomass output of the plants (Munawar et al., 2019). Moreover, supplementation of both NO and NOSH (donor of H_2_S) significantly alleviates the drought stress in *Medicago sativa* via stimulation of major antioxidant genes (*GST17*, *Cu/ZnSOD*, *FeSOD*, *cAPX*), linked with ROS scavenging (Antoniou et al., 2020).

#### 3.1.2 NO under salinity stress

Salinity in soil hinders major physiological processes in plants. Salinity stress causes lipid peroxidation, changes the Na^+^/K^+^ ratio, and reduces the transport of essential elements, distorting plant structure and growth. Nitric oxide synthase (NOS) is the key enzyme of the NO biosynthetic pathway, particularly expressed in abiotic stress. It is noted that unfavourable conditions induce endogenous NO production mainly through the highly expressed NOS1 enzyme and nitrate reductase (NR) pathways. Accumulation of NO in plants alleviates salt stress as it increases isoflavonoid synthesis and related gene (*CHR*, *CHIA*, *C4H*, and *IFS*) expressions (Yin et al., 2022). The application of SNP in the case of *Panax ginseng* and rice (*Oryza sativa* L.) plants significantly boosted the activity of antioxidant enzymes, such as SOD, CAT, APX, POX, and polyphenol peroxidase (PPO), to protect the plant from salt stress (Rahim et al., 2022). Similarly, in the case of *Brassica napus* L., NaCl exposure in plants causes phytotoxicity; however, NO treatment controls the toxic effects and improves phenolic content and PAL enzyme activity (Karimi et al., 2020). Moreover, exogenous supplementation of SNP to *Silybum marianum* L. seedlings enhances the therapeutic properties of phenolics and flavonoids (Zangani et al., 2018).

#### 3.1.3 NO under heavy metal stress

Many studies stated that plants upon exposure to Heavy metals (HMs) stress exhibit ultrastructural changes, including chlorosis, disorganized photosynthesis system, root and shoot damage, and senescence. Heavy metals stimulate the plants’ endogenous SNP production. Application of SNP stimulates SMs production to resist metals (Pb and Cd)/metalloid effects, resulting in improved shoot and root dry weights, and growth indices in various plant species (Emamverdiansss et al., 2021). Under Cd stress, exposure of SNP (200µM) in *Artemisia annua* enhances the artemisinin (present in glandular secretory trichomes) content (Wani et al., 2023). Under stress conditions, the production of phenolic compounds decreases; however, the application of exogenous NO increased anthocyanins, flavonols, and flavonoids in seedlings of *Solanum tuberosum* (Nabati et al., 2024). Moreover, above the permissible limit, copper (Cu) shows toxic symptoms in plants, such as necrosis and growth retardation. In *Raphanus sativus*, Cu toxicity is overcome by supplementing NO (200 µM), resulting in enhanced phenolic, flavonoid, and anthocyanin concentrations to reduce free radicals (Bhardwaj et al., 2023). Overall, research shows that NO’s role becomes more pronounced in regulating the production of metabolites to alleviate stress in plants.

## 4 Role of hydrogen sulfide in SM production under stress conditions

Hydrogen sulfide is primarily known as a gaseous transmitter and has been recognized to play a vital role in cellular and physiological processes in plants. It involves cell proliferation, seed germination, stomatal conductance, root formation, overall plant development, and enhancing plant responses to environmental stresses (Zhang et al., 2020). Hydrogen sulfide is a highly mobile and transported molecule throughout the plants. Sub-cellular compartments, including the chloroplast, cytosol, and mitochondria, serve as the sites for H_2_S formation, where sulfur and cysteine metabolism-related enzymes drive its biogenesis (Zhou et al., 2020). Hydrogen sulfide and its donors (NaHS) impact SMs by influencing sulfur metabolism and modulating stress responses to counteract by eliminating ROS, thereby maintaining intracellular redox balance (Rizwan et al., 2019). It is reported that numerous H_2_S donors, such as sodium hydrogen sulfide (NaHS), calcium sulfide, and diallyl trisulfide (DATS), also participate under abiotic stress (Hilal et al., 2023). Hydrogen sulfide also positively affects the postharvest storage of vegetables and fruits. In tomatoes (*Solanum lyocpersicum* L.), it raised the level of SM and their regulation via high gene expression. Moreover, H_2_S increases the expression of *PAL5*, *monodehydroascorbate reductase (MDHAR)*, and *dehydroascorbate reductase 1* (*DHAR1*) genes along with flavonoid-related genes like *chalcone synthase 1 or 2*(*CHS1 or 2*)*, flavanone 3-dioxygenase* (*F3H*), and *flavanol synthase* (*FLS*) (Zhang Y. et al., 2023). Besides, under Cd^2+^ stress, the photosynthetic system in *Vigna radiata* tends to be repaired by exogenous NO and H_2_S application, with Ca^2+^, which acts as an intermediate (Khan et al., 2020). These studies highlight the potential role of H_2_S in enhancing SMs synthesis under abiotic stress (**Table 2**).

**Table 2:** Exploring the role of sodium hydrogen sulfide (NaHS) in managing secondary metabolite production in diverse plant species under abiotic stress.

Abiotic stress	Experimental plants	Concentration of NaHS (H_2_S donor)	Alteration in secondary metabolite production	References
Drought	*Carthamus tinctorius*	0.5 and 1mM NaHS	Secondary metabolites production↑, ion homeostasis ↑, and antioxidant capacity↑.	(Amir et al., 2021)
	*Triticum aestivum*	100-500 µM NaHS	MDA concentration↓, anthocyanin content↑, proline level ↑, and flavonoid accumulation ↑.	(Kolupaev et al., 2019)
Salt	*Cyclocarya paliurus*	0.5 mM NaHS	Total flavonoid↑, kaempferol-3-O-glucoside ↑, kaempferol-3-O-rhamnoside↑ and quercetin-3-O rhamnoside↑.	(Zhang L. et al., 2023)
	*Fragaria×ananassa* Duch.	200 µM NaHS	Biosynthesis of phenylpropanoid ↑, Anthocyanin content ↑, cell proliferation ↓, and ROS formation ↓.	(Pourebrahimi et al., 2023)
	*Zea mays*	0.5 mM NaHS	Polyphenol oxidase (PPO) activity ↑, antioxidant activity ↑, and oxidative stress ↓.	(Shoukat et al., 2023)
	*Satureja hortensis*	0.2 mM NaHS	Oxygenated monoterpenes such as Carvacrol↑, p-cymene↑, γ-terpinene↑, and α-terpinene↑.	(Khalofah et al., 2024)
Heavy metal	*Artemisia annua*	200 µM NaHS	Artemisinin content↑, antioxidant accumulation ↑, and ROS species formation ↓.	(Nomani et al., 2022)
	*Matthiola incana*	1.5 mM NaHS	PAL activity↑, polyphenol oxidase (PPO) ↑, and antioxidants ↑.	(Zulfiqar et al., 2024)
	*Zea mays*	NaHS	Phenolic content ↑, flavonoid content↑, and proline accumulation ↑ in vacuoles.	(Alatawi et al., 2023)

↑, Increase; ↓, Decrease.

### 4.1 H_2_S under salinity stress

High accumulation of various salts in the soil produces osmotic stress and ion toxicity, which negatively regulate nutrient uptake, physiological activity, and metabolic pathways. As a result, the accumulation of salinity in plants increases ROS at the subcellular level. Foliar spray of NaHS in batal (*Cyclocarya paliurus*) leaves escalates phenolic content and many other chemical constituents such as quercetin-3-0-rhamnoside and kaemferol-3-0-rhamnoside, and 3-O-caffeoylquinic acid (Chen et al., 2021). As stated, phenolic compounds, equipped with a hydroxyl group, can lessen the toxicity of ROS. Alamer (2023) demonstrated that H_2_S has a beneficial effect in wheat (*Triticum aestivum* L.) crops upon applying it and upgrades PAL enzyme activity, resulting in an improved phenolic level compared to control and NaCl-treated plants. Other research proved that *Satureja hortensis* treated with 0.2 mM NaHS increases oxygenated monoterpenes such as carvacrol, p-cymene, γ-terpinene, and α-terpinene under high saline conditions (Khalofah et al., 2024).

### 4.2 H_2_S under heavy metal stress

Heavy metal stress correlates with ROS accumulation and antioxidant formation in response to oxidative injury. When exposed to HM stress, SMs like flavonoids and anthocyanins significantly diminish redox imbalance within plant cells (Maleki et al., 2017; Goncharuk and Zagoskina, 2023). Under arsenic (As) stress, H_2_S and melatonin (MT) supplementation in tomato seedlings increases antioxidant phenolic compounds (anthocyanin, polyphenols, and flavonoids), helping reduce oxidative stress (Ghorbani et al., 2024). Besides phenolics, terpenoids also help in mitigating abiotic stress in cucumbers (*Cucumus sativa* L.) under chilling conditions. The tetracyclic triterpenoid known as cucurbitacin protects plants from both abiotic and biotic stress. This protective effect is notably enhanced when plants are treated with NaHS (Liu Z. et al., 2019). A similar study was conducted on *Cucurbita pepo* under nickel (Ni) stress with supplementation of various concentrations (50, 100, 200, and 400 μM) of NaHS, under normal conditions. Whereas, among all the applied doses, 100 μM of NaHS improved phenolics, anthocyanins, and sinigrin (glucosinolate) content in roots and aerial parts (Valivand and Amooaghaie, 2021a). Besides, combined supplementation of NO and H_2_S to form nitrsothiols, which significantly increases resistance against pathogenic attack (Corpas et al., 2024). Although the application of NaHS and SNP regulates the endogenous production of NO and protects Bermuda grass from lead toxicity (Shi et al., 2014).

## 5 Role of methyl jasmonate in SM production under stress conditions

Jasmonic acid and its derivatives (MeJA and Ja-Ile) are collectively known as jasmonate. Methyl jasmonate is highly volatile and aids in controlling a variety of abiotic and biotic stress. To overcome the penalties of environmental stress, it stimulates molecular signal transduction and gene expression regulation, which leads to the accumulation of SMs (Jeyasri et al., 2023). It induces the gene expression involved in alkaloids, phenolics, and terpenes biosynthesis to improve the plant’s survival abilities. Several transcription factors, including WRKY, bZIP, basic-helix-loop-helix (bHLH), NAC, ERF, MYB, and MYC2 families, regulate the MeJA signalling process, as well as upregulate SM production (Kumar et al., 2021). These transcription factors are essential for regulating the synthesis of compounds like flavonoids and phenols. For instance, in *Salvia miltiorrhiza*, the MeJA-responsive MYB factor stimulates phenolic acid accumulation (Zhou W. et al., 2021). A similar role of WRKY1 in *Artemisia annua* is that it regulates artemisinin biosynthesis by binding to the promoter of the sesquiterpene synthesis gene. According to Davari et al. (2018), *Satureja hortensis* treated with JA shows a considerable improvement in the quantities of α-pinene and monoterpene hydrocarbons in essential oil. Further, exogenous MeJA enhanced the expression of disease-resistance genes like *RrRGA3*, *RrPPO*, *RrCHIT*, *RrPRB1*, and *RrRPM1*. It also activated genes related to AsA biosynthesis and the AsA–GSH cycle, including *RrMIXO*, *RrAKRC9*, *RrDHAR*, and *RrGPX*, leading to an increase in AsA levels. Methyl jasmonate amplifies activities of PAL, C4H, 4CL enzymes, and gene expression (*RrPAL*, *Rr4CL*, *RrCSE*, *RrCCR*, *RrPGT*, *RrHCT*, *RrDFR*, *RrERF114*) in the phenylpropanoid pathway. This upregulation resulted in a higher accumulation of L-phenylalanine, caffeic acid, phlorizin, other phenolic compounds, and lignin in *Rosa roxburghii* (Ma et al., 2025).

### 5.1 MeJA under drought stress

Drought stress affects regular activities and manifests detrimental symptoms in stressed plants. Plants stimulate flavonoid biosynthesis, which sustains antioxidant characteristics to protect plants. Several *in vitro* studies have shown that drought stress is interrelated with SM accumulation in plants and associated with metabolic activities (Ghosh et al., 2018). Genome editing in SM regulatory genes can improve drought tolerance according to their functions. For example, bHLH transcription factors exist in various plant species and synchronize with flavonoid biosynthesis to improve drought effects. *VvbHLH1* (members of the bHLH family) overexpressed in *Arabidopsis* and modulates phenolic/flavonoid biosynthesis, ABA signalling, osmolytes production, and ROS scavenging systems (Wang F. et al., 2016). For example, the application of MeJA increases SMs (anthocyanin, phenolics, and carotenoids), which act as superoxide and hydroxide radical scavengers and deplete drought stress in *Ocimum basilicum* (Lopes et al., 2024). MeJA administration enhances the total phenolic content in peppermint leaves (Shenavaie Zare et al., 2022). Furthermore, soybeans (*Glycine max* L.) under drought stress have shown a reduction in total flavonoid content, but MeJA supplementation increases the accumulation of isoflavonoids and phenolic compounds (Mohamed and Latif, 2017). Under water deficit conditions in *Astragalus membranaceus*, treatment of 200µM MeJA improved glycoside (calycosin-7-*O*-β-d-glucoside) accumulation with diverse therapeutic properties (Feng et al., 2021). Aromatic amino acids act as precursors for SM synthesis, such as phenylalanine and tyrosine, and participate in flavonoid accumulation during adverse conditions. Glucosinolates derived from sulfur-containing amino acids (methionine, tryptophan) are well-known for providing tolerance against biotic and abiotic stress. Tang et al. (2023) confirmed that applying MeJA in *Prunella vulgaris* L. enhanced the activity of PAL, tyrosine aminotransferase (TAT), and 4-coumaroyl CoA ligase (4-CL) enzymes that are involved in the phenylpropanoid pathway. The activation of these enzymes helps in improving membrane stability and photoprotection machinery in plants.

### 5.2 MeJA under salinity stress

Soil salinization is the most significant agricultural sector issue, particularly in dry and semi-arid areas. The presence of salt stress generates ROS, which can degrade both carotenoids and secondary compounds. Interestingly, MeJA treatment improves the photoprotection mechanism via the accumulation of carotenoids and regulates antioxidants (SOD, POX, DHAR, and CAT) to overcome cellular oxidative stress. Kiani et al. (2021) verified that 60 µM of MeJA enhanced the photosynthetic pigment (chlorophyll and carotenoids) and also modulated phenolic compounds. He et al. (2021) also demonstrated that higher concentrations of NaCl reduce carotenoid content in *Zea mays*, following MeJA supplementation, the amount of carotenoid, lutein, and zeaxanthin rises in NaCl-stressed plants. Several studies focused on the influential characteristics of MeJA in SM production, particularly members of the Lamiaceae family under saline toxicity. For instance, *Mentha piperita* undergoes treatment of MeJA and shows an elevation in methyl acetate, β-pinene, and 1,8-cineole (Afkar and Karimzadeh, 2014). Saeed et al. (2017) experimented with the medicinal plant *Ajuga bracteosa* to explore the impact of MeJA and phenylacetic acid (PAA) on its growth parameters and bioactive constituents. They observed that MeJA and PAA application increased the total phenolic (lignin and tannin) and flavonoid content in the root suspension of *A. bracteosa.* Previous studies documented that MeJA treatment facilitates the biosynthesis of SMs in plant cells by influencing ROS scavenging activities and activating pivotal antioxidant genes (Ho et al., 2020; Khan et al., 2024a). Furthermore, MeJA has been extensively deployed as an elicitor in various species of medicinal plants to increase the production of SMs in the cell culture system. The combined application of salicylic acid (SA) and MeJA upgrades flavonoids and phenolic yield in *Phyllanthus pulcher* (Danaee et al., 2015). A similar study highlights the elicitation potentiality of MeJA and SA in the case of *Cuminum cyminum*, where MeJA dominates the involvement of key genes and enzymes involved in phenolic compound synthesis (Rahimi et al., 2013). They suggested that this stimulation boosts cellular activities at both biochemical and molecular levels through various signalling compounds. Additionally, MeJA plays a decisive role in signal transduction, accelerating enzyme catalysis, and promoting the production of specific compounds (polyphenols, terpenoids, flavonoids, and alkaloids) (Rahimi et al., 2013; Ho et al., 2020). **Table 3** summarizes the effects of MeJA treatment on the concentrations of flavonoids, polyphenols, and isoprene compounds (volatile and non-volatile) in a toxic environment.

**Table 3:** Exploring the role of methyl jasmonate (MeJA) in managing secondary metabolite production in diverse plant species under abiotic stress.

Abiotic stress	Experimental plants	Concentration of MeJA	Alteration in secondary metabolite production	References
Drought	*Brassica juncea*	MeJA	Carotenoid content ↑, anthocyanin ↑, total phenolic, and flavonoid content ↑.	(Khan et al., 2024b)
	*Foeniculum vulgare*	75 µM MeJA	Activation of phenylpropanoid pathways ↑, phenolic content↑, e-anethole, and fenchone↑.	(Peymaei et al., 2024)
	*Prunella vulgaris*	1.0 Mm MeJA	Total phenolics ↑, total flavonoids ↑, rosmarinic acid, and hyperoside content ↑.	(Tang et al., 2023)
	*Andrographis paniculata*	250 µM MeJA	Diterpenoids, such as neoandrographolide ↑, 14-deoxyandrographolide ↑, and andrographolide ↑.	(Chungloo et al., 2023)
	*Portulaca oleracea*	1.0 mM MeJA	Phenolic compounds, such as bergenin ↑, aconitic acid ↑, miserotoxin ↑ 1- monoplamitin ↑.	(Nyanasaigran et al., 2024)
Salt	*Glycyrrhiza uralensis*	30 µM MeJA	Total flavonoid content ↑, total saponin content↑, total anthocyanin content ↑, glycyrrhizin ↑, and licochalcone↑.	(Ma et al., 2023)
	*Anchusa italica*	60 µM MeJA	Total phenolic content ↑, total soluble sugar ↑, and proline accumulation ↑.	(Taheri et al., 2020)
	*Ocimum basilicum*	0.5 mM MeJA	Monoterpenoids such as Linalool ↑, 1,8-cineole, Content ↑.	(Talebi et al., 2018)
Heavy metal	*Cajanus cajan*	10 and 100 mM MeJA	Phenolics, such as Pyrogallol, caffeic acid, resorcinol content ↑, and flavonoids, naringenin, catechol, gossypin, and quercetin content ↑ under Cd stress.	(Kaushik et al., 2024)
	*Solanum lycopersicum*	5 µM MeJA	The activity of phenyl ammonia lyase (PAL) ↑, Phenolic content, Antioxidants (SOD, CAT, and POX) ↑, and ROS accumulation ↓ under Cd stress.	(Qin et al., 2024)
	*Oryza sativa*	MeJA	Anthocyanin content ↑, photooxidation↓, and antioxidant accumulation↑ under Cr toxicity.	(Zhang Q. et al., 2022)
	*Cymbopogon flexuosus*	MeJA	α-tocopherol content ↑, β-carotene accumulation↑, and proline concentration ↑during As stress.	(Saleem et al., 2023)

↑, Increase; ↓, Decrease.

### 5.3 MeJA under heavy metal stress

Plants face HMs toxicity, such as chromium (Cr), Cd, arsenic (As), and Cu in the soil that are taken up by plants through the transporters, altering physiological, biochemical, and genetic compositions (Ahmad et al., 2018). In exposure to HMs, phenols act as efficient chelators. Plant root secretes phenolic compounds (p-hydroxybenzoic and vanillic) and flavonoids (naringenin and quercetin) that help in the solubilization of metals in the soil (Quartacci et al., 2009). Additionally, antioxidants regulate ROS levels and are crucial for developing heavy metal tolerance strategies in plants. Polyphenols, terpenes, and various vitamins play a key role in downregulating O_2_^•–^ and minimizing the effect of oxidative imbalance. An exogenous supply of MeJA under As stress significantly alleviates the toxicity and further amplifies the PAL and polyphenol oxidase (PPO) enzyme activities (Farooq et al., 2016). One of the antioxidants, e.g., astaxanthin (tetraterpenoids) obtained from *Haematococcus pluvialis* highly accumulated when exposed to MeJA, causes the astaxanthin-related genes (*PSY, BKT, and CRTR-b*) to express, for its high accumulation (Liu Y. et al., 2019). In a study, the supply of MeJA to *Centella asiatica* increased the concentration of triterpenoids via increment in PAL enzyme and antioxidant enzyme activity (Buraphaka and Putalun, 2020). Under selenium (Se) stress, the species *Plantago ovata* was exposed to JA (10 µM), which synergistically improved the provision of numerous SMs. For instance, hydroxycinnamic acids, flavonoids, lignins, tannins, and other similar compounds serve as facilitators in stress responses (Dey and Raychaudhuri, 2024).

## 6 Role of hydrogen peroxide in SM production under stress conditions

In recent years, considerable attention has been paid to H_2_O_2_, which acts as a signalling molecule, triggering various physiological and biochemical responses in plants. Hydrogen peroxide signals trigger adaptive responses affecting cell proliferation, differentiation, transportation, plant survival, and numerous metabolic processes. These physiological processes include seed germination, seedling maturation, stomatal movement, photosynthesis, cell growth and development, antioxidant systems, and senescence, which is significantly regulated by H_2_O_2_ (Nazir et al., 2020; Fatima et al., 2023). Hydrogen peroxide is a free radical belonging to the ROS family, synthesized from three main routes: photorespiration, electron transport series, and redox reaction in apoplast (Černý et al., 2018). It can initiate pathways that lead to the synthesis of SMs, which often help plant overall resilience, as mentioned in **Table 4**.

**Table 4:** Exploring the role of hydrogen peroxide (H_2_O_2_) in managing secondary metabolite production in diverse plant species under abiotic stress.

Abiotic stress	Experimental plants	Concentration of H_2_O_2_	Alteration in secondary metabolite production	References
Drought	*Triticum aestivum*	50 μM H_2_O_2_	Leaf osmotic potential ↑, leaf water content ↑, MDA content↓, and antioxidant enzyme activity ↑.	(Sehar et al., 2021)
	*Sorghum bicolor*	100 mM H_2_O_2_	Stabilizing the cell membrane↑, photosynthetic rate ↑, and ROS formation ↓.	(Song K. E. et al., 2023)
	*Hordeum vulgare*	5 mM H_2_O_2_	SOD↑, CAT↑, APX↑, and POX↑, lipid peroxidation ↓, secondary metabolites accumulation ↑.	(Skowron and Trojak, 2021)
Salt	*Ocimum bacilicum*	2.5 mM H_2_O_2_	Anthocyanin content ↑, APX↑, and bioactive constituents ↑.	(Gohari et al., 2020)
	*Lactuca sativa*	0.1 mM H_2_O_2_	Root shoot growth ↑, Carotenoid content ↑, photosynthetic efficiency ↑.	(Silva et al., 2023)
Heavy metal	*Triticum aestivum*	30 µM H_2_O_2_	Seed germination ↑, stomatal conductance ↑, proline accumulation ↑, and total chlorophyll content ↑ under Cu toxicity.	(Alhammad et al., 2023)
	*Brassica napus*	50 μM H_2_O_2_	SOD↑, CAT↑, APX↑ and POX↑, MDHAR and DHAR activities↑, GR activity ↑ under Cd toxicity.	(Hasanuzzaman et al., 2017)

↑, Increase; ↓, Decrease.

### 6.1 H_2_O_2_ under drought stress

The production of ROS under drought stress modulates plant metabolic machinery in mitochondria, cytoplasm, and peroxisome, and damages cell basic elements (lipids, proteins, and carbohydrates) (Nadarajah, 2020). Applying H_2_O_2_ in plant species positively influences their tolerance mechanisms. Hydrogen peroxide modulates the PPO activities by oxidising phenolic compounds associated with antioxidant activity. Under drought stress conditions, pre-treatment of H_2_O_2_ influences POX and PPO working mechanisms, ultimately improving phenolic content in root and shoot cells (Bhardwaj et al., 2021). Hydrogen peroxide treatment in soybeans increases the PAL enzyme activity, which boosts total phenolic accumulation compared to stress-affected plants (Darmanti et al., 2021). H_2_O_2_ (10 μM) applied on wheat plants reveals a significant elevation in osmolytes, physiological attributes, K^+^ accumulation, membrane stability, and reduced ROS formation (Singh et al., 2021). Using a metabolomic approach, leaf metabolites in maize were modulated by 10 mM of H_2_O_2_ and salinity. Specifically, 42 of the 51 metabolites remain unchanged in plants treated with H_2_O_2_ under non-saline conditions (Dos Santos Araújo et al., 2021).

### 6.2 H_2_O_2_ under salinity stress

Soil salinity is a prominent cause of land deterioration after soil erosion has posed persistent challenges to agriculture. Hydrogen peroxide influences various physiological processes, including stomatal closure, root growth, and nutrient uptake, that support plant adaptation to saline environments. The seed priming with 5 mM H_2_O_2_ under saline toxicity enhances ferulic, hesperidin, quercetin, luteolin, and rosmarinic acid in *Salvia officinalis* (Amooaghaie et al., 2024). Further, lettuce (*Lactuca sativa* L.) plants exposed to H_2_O_2_ exhibit increased levels of phenolic compounds that provide antioxidant effects attributed to the hydroxyl group in the benzene ring. It revives many structural genes (*PAL, DFR, UFGT*, and *CHS*) of phenylpropanoid metabolism (Wang et al., 2023). Sodium nitroprusside and H_2_O_2_ are the priming agents that increase plant tolerance against a range of abiotic stress. The combined treatment of 200 μM SNP + 2.5 mM H_2_O_2_ effectively alleviated salinity stress in *Ocimum bacilicum* via an increment in anthocyanin, APX activity, Chl a and b, and key metabolites like methyl chavicol, linalool, cadinol, and epi-α-cadinol (Gohari et al., 2020). Similarly, NO and H_2_O_2_ in *Brassica napus* L. during saline stress increased antioxidant activity and stimulated PAL activity, which influences flavonoid content (Karimi et al., 2020). Seeds of *Brassica oleracea* var. *botrytis* primed with H_2_O_2_ improved the defence mechanism and reduced lipid peroxidation and electrolyte leakage (Ellouzi et al., 2021). Further, foliar application of H_2_O_2_ helps to adapt strategies that minimize saline toxicity in cotton plants by providing strength to the antioxidant system (Nóbrega et al., 2024).

### 6.3 H_2_O_2_ under heavy metal stress

To overcome the consequences of HMs, H_2_O_2_ strengthens the defence machinery in plants to balance the regulatory function. For instance, under As toxicity, 50 μM H_2_O_2_ is applied in *Oryza sativa*, augmenting carotenoid content, osmolytes (proline) in vacuoles and SOD, CAT, and POX activities (Asgher et al., 2021). Controlled application of H_2_O_2_ can potentially boost metabolite levels during cultivation. In *Aquilaria sinensis*, the early response to wound stress involves the activation of *AsTPS10, AsTPS16*, and *AsTPS19*, which initiate a H_2_O_2_ signalling pathway that leads to the accumulation of sesquiterpenes (Lv et al., 2017). Similarly, in *Ficus deltoidei*, the application of H_2_O_2_, from the vegetative stage to the flowering stage, also influences the plant to generate antioxidant enzymes (POX and PPO) for rapid ROS scavenging (Nurnaeimah et al., 2020). Nazir et al. (2019) reported that H_2_O_2_ root dipping treatment had a significant and positive effect on growth and yield characteristics. Besides, following treatment, the leaf’s potential to retain water, photosynthetic pigments, stomatal movement, antioxidant system, and osmoprotectant improves against Cu stress.

## 7 Role of ethylene in SM production under stress conditions

Ethylene (ETH) is one of the key signalling molecules in plant development (seed germination to leaf abscission) and SM production during stress conditions. Ethylene receptors are predominantly present on the endoplasmic reticulum (ER) and are negatively regulated by various factors such as ETR1, ETR2, ERS1, ERS2, and EIN4. Besides, the AP2/ERF (ethylene-responsive elements) factor notably regulates stress-responsive genes, including *Dehydrin, LEA, HMA, ALMT, SERP2, RLK*, and *MAPKKK3*, which are recognized for controlling abiotic and biotic stress (Feng et al., 2020). Ethylene positively influences the number of SMs (phenolics, terpenoids, and alkaloids) in plants which provide resistance against biotic and abiotic stress. Ke et al. (2018) postulated that ETH modulates flavonol biosynthesis by regulating the expression of chalcone isomerase, CHS, and flavonol synthase via the MYB12 transcription factor. Further, ETH precursor (1-aminocyclopropane-1-carboxylic acid, ACC) also participated in phenolic synthesis via high expression of anthranilate synthase (AS), PAL, and isochorismate synthase (ICS) in plants (Liu et al., 2016).

### 7.1 ETH under drought stress

Different plant species experience drought to varying degrees, and they frequently coincide with a temperature rise. To combat drought stress, plants produce a class of SMs that reduce ionic imbalance and ROS toxicity and maintain the interrelation between phenotype and genotype of the plant (Salvi et al., 2021). Knowing the function of ETH can aid in creating plants to increase the synthesis of SM in crops during droughts. ETH plays a dual role under drought stress as a prooxidant (ROS accumulator) and antioxidant (ROS scavenger). It was noticed that applying ethephon (a diverse form of ETH) to the seeds elevates antioxidants and osmolyte production and lessens lipid peroxidation (Zhang et al., 2016; Kowsalya et al., 2025). Furthermore, ERF also controls root development and maturation under drought stress. Kumar et al. (2022) reported that under drought stress, overexpressing the *OsAP2/ERF-N22* line leads to elevated stomatal conductance, transpiration movement, improved membrane stability index, higher relative water content, and osmotic potential.

### 7.2 ETH under salinity stress

Signalling molecules are essential for managing stress, providing tolerance, and positive regulation of plant metabolic activities throughout the life cycle. ROS generally determines how ETH reacts downstream under stress conditions because ETH signalling depends on ROS levels. Endogenous and exogenous supply of ETH-releasing compounds (ethephon and ACC) reduces salinity in diverse plant species (Iqbal et al., 2017). Applying ethephon in the *Daucus carota* root to accumulate more anthocyanin via raising their structural genes is likely driven by the effect of *DcMYB1*’s influence on the *DcPAL* gene (Barba-Espín et al., 2017). Furthermore, elevated ETH production or persistent activation of ETH signalling resulted in reducing ROS accumulation in root vascular tissue, which contributes to improved salt tolerance. Ethephon increases phenolic and phenylpropanoid content in *Fagopyrum esculentum* during high saline conditions. Phenylpropanoid provides better resistance against various types of stress, including water, UV, and wound stress (Li et al., 2017).

### 7.3 ETH under heavy metal stress

Ethylene helps plants cope with the adverse effects of HMs by modulating stress signalling, enhancing antioxidant production, and facilitating metal sequestration. *Arabidopsis* treated with zinc oxide (ZnO) nanoparticles, ETH signalling deficient mutants *ein2–1* and *etr1–3* exhibited higher SOD, CAT, APX, and POX activity compared to wild-type plants (Khan et al., 2019). Under Cd toxicity, *Lycium chinense* up-regulates the *LchERF* gene and GSG accumulation, which increases the production of ETH and helps overcome Cd toxicity (Guan et al., 2016). Similarly, the supplementation of ETH in Cd-stressed *Catharanthus roseus* impedes ROS and MDA production and promotes the accumulation of metallothionein (a cysteine-rich biomolecule) (Chen et al., 2017). An experimental study revealed that As-tolerant mutant *eto1–1* has a high production of phytochelatins (PCs) as compared to the wild type, proving that ETH better responds in *Arabidopsis thaliana* under metal stress (Zou et al., 2024). Subsequent research also reported that, in comparison to the wild-type, the ETH-producing mutant *eto1–1* produced less MDA and O_2_^•–^ during As toxicity. Conversely, the application of 100 µM of ACC to *Nelumbo nucifera* G. under Cd exposure increases antioxidant activities, traps oxidative radicles, and reduces MDA and electrolyte leakage (Wang et al., 2020). **Table 5** illustrates various examples of plant species under abiotic stress, where supplementation of ETH increases SMs.

**Table 5:** Exploring the role of ethylene (ETH) in managing secondary metabolite production in diverse plant species under abiotic stress.

Abiotic stress	Experimental plants	Form and concentration of ethylene	Alteration in secondary metabolite production	References
Drought	*Triticum aestivum*	200 mg kg^−1^ ethephon	Caffeate O-methyltransferase and peroxidase ↑, phenylpropanoid synthesis ↑, root volume, and leaf area ↑.	(Yang et al., 2021)
Salt	*Fagopyrum tataricum*	0.4- and 0.6-mM ETH	MDA content ↓, photosynthetic pigments Chl *a* and Chl *b*↑, and ion balance ↑.	(Xu et al., 2022)
	*Oryza sativa*	Ethephon	Seedling growth ↑, ROS toxicity ↓, antioxidant enzyme activity↑, and MDA content ↓.	(Hussain et al., 2020)
	*Brassica juncea*	200 µL L^−1^ ethephon	Photosynthetic pigment ↑, intracellular CO_2_ ↑, and antioxidant enzyme activity ↑.	(Rasheed et al., 2021)
Heavy metal	*Brassica juncea*	200 µl L^−1^ ethephon	Seed germination↑, chlorophyll content ↑, SOD ↑, CAT↑, POX↑, and APX↑ under Cr stress.	(Asgher et al., 2023)
	*Brassica juncea*	200 μL L^−1^ ethephon	ROS ↓, reduced GSH ↑, PSII activity ↑, antioxidant capacity ↑, and photosynthetic potential ↑.	(Khan and Khan, 2014)

↑, Increase; ↓, Decrease.

## 8 Role of melatonin in SM production under stress conditions

Phytomelatonin (*N*-acetyl-5-methoxy tryptamine, MT) is an indole compound derived from serotonin and is an emerging molecule that regulates diverse functions in plants, including osmo protectant, seed germination, photosynthesis, and delayed senescence (Zhang T. et al., 2022). Plants have two main pathways for synthesising phytomelatonin: primary and secondary pathways. The main enzymes involved in the synthesis of MT in plants are tryptophan hydroxylase (TPH), caffeic acid o-methyltransferase (COMT), tryptophan decarboxylase (TDC), and serotonin N-acyltransferase (SNAT) (He et al., 2023). In the case of plants, SNAT is found within the chloroplast, facilitating the conversion of serotonin to melatonin, whereas in vertebrates, MT production takes place in mitochondria. Melatonin supplementation in plants significantly reduced ROS-generated stress via antioxidant formation and synthesis of SMs (Arnao et al., 2022). Melatonin behaves like an efficient free radical scavenger for OH, H_2_O_2_, peroxynitrite anion (ONOO^-^), alkoxy radical (RO), and peroxyl radical (ROO). Moreover, MT induces a diversity of compounds including terpenoids (mono, di, tri, and polyterpenes), phenolics, anthocyanins, flavonoids, and glucosinolates. It improves the post-harvest quality of fruits and vegetables by the activation of crucial enzymes such as PAL, CHS, ANR, DFR, and OMT. This enzyme upregulates under stress conditions and accumulates phenolics and flavonoid amounts in plants (Li et al., 2020).

### 8.1 MT under drought stress

One of the main consequences of drought stress in plants is a reduced CO_2_ assimilation rate that energizes the overproduction of ROS, which causes impairment at the cellular level. Melatonin application stabilizes the defence system of plant species by promoting the production of SMs and antioxidants (Nguyen et al., 2022). For example, 50 μM of MT treatment under drought conditions increases the anthocyanin content in *Helianthus annuus* L. It is also linked with regulating phenolics and flavonoids in plants, where these metabolites act as ROS scavengers (Mahmood et al., 2024). In a related finding, *Taxus baccata* L. shows that a 100 μM concentration of MT improves phenolic content, SOD, CAT, and POX activities, thus enhancing plant resilience. Melatonin also stimulates the expression of taxol biosynthetic genes (*TXS and DBAT*), which proves that MT is a modulator of gene expression in both contexts (Shahmohammadi et al., 2024). A positive role of MT (200 μM) under drought conditions maximizes the accumulation of carotenoids and phenolic compounds in *Brassica rapa* (Hasnain et al., 2023).

### 8.2 MT under salinity stress

Melatonin works as a stress- reliever alleviating H_2_O_2_, inducing the Na^+^/K^+^ pump, and stimulating ROS catabolizing enzymes. According to Sheikhalipour et al. (2022), MT influences *WRKY, SOAR1*, and *Mc5PTase7* genes, which provide stress tolerance and modulate secondary metabolism. These alterations were proceeded by *MAP30, α-MMC*, and *PAL* genes, which were expressed in the stressed and MT-treated plants of *Momordica charantia* L. under saline conditions. Under salt stress, *Trigonella foenum- graecum* L. treated with different doses of MT, i.e., 60 and 90 mg L^-1^, increased alkaloid content by activating *Squalane synthase* (*SQS)* and *CAS* gene, which is pivotal in diosgenin biosynthesis. Additionally, MT improved the levels of phenolics and flavonoids to maintain ion homeostasis (Mohamadi Esboei et al., 2022). MT positively affected the activity of flavonoids in plants facing various abiotic stress conditions. For example, MT treatment in grape berries (*Vitis vinifera* L.) upscale total phenol compounds, total flavonoids, and DPPH, and upregulates the expression of the *PAL*, *STS*, *4CL*, and *CHS* genes, enhancing the accumulation of flavanones, flavanols, and flavonoids (Xu et al., 2017). Similarly, Jahan et al. (2020) explained that MT supplementation increases CHS enzyme activity under stress conditions, whereas CHS plays a role in anthocyanin production. Besides, co-exposure of MT and *Pseudomonas fluorescens* in *Brassica juncea* enhances the production of notable flavonoids, specifically kaempferol, cyanidin, naringenin, quercetin, and myricetin. These flavonoids are vital for safeguarding cells that engage in photosynthesis (Khan et al., 2023).

### 8.3 MT under heavy metal stress

Various signalling molecules efficiently improve photosynthetic apparatus, growth metrics, and SM biosynthesis to combat HM toxicity (Aftab and Roychoudhury, 2021). Foliar treatment of MT in strawberries (*Fragaria x ananassa*) against Cd (1mM) stress increases anthocyanin and phenolic enzymatic activities, and decreases lipid peroxidation and electrolyte leakage (Saqib et al., 2023). Conversely, the application of MT (100 μM) in the case of the *Brassica napus* plant under cobalt stress improved the expression of genes involved in SM metabolism, such as *PAL, PPO*, and *CAD* in roots and leaves, which markedly reduced oxidative stress (Ali et al., 2023). In *Camellia sinensis*, catechin is a major SM, which is enhanced in the presence of MT under As stress. MT elevates the transcript level of anthocyanin and catechin genes (*CsCHS, CsCHI, CsF3H, CsDFR*, and *CsANS*), which significantly increases metal tolerance (Li et al., 2021). Dou et al. (2022) also reported that an exogenous supply of MT in *Solanum lycopersicum* escalated 3 flavonoids (quercetin, rutin, and naringenin) and six phenolic compounds (caffeic acid, p-hydroxybenzoic acid, protocatechuic acid, chlorogenic acid, gentisic acid, and sinapic acid) to combat metal toxicity. **Table 6** reveals that the MT application under various abiotic stresses alters secondary metabolite production and mitigates stress toxicity.

**Table 6:** Exploring the role of melatonin (MT) in managing secondary metabolite production in diverse plant species under abiotic stress.

Abiotic stress	Experimental plants	Concentration of MT	Alteration in secondary metabolite production	References
Drought	two citrus cultivars, viz., *C. latifolia* and *C. aurantifolia*	50 and 100 µM MT	Polyphenols ↑, non-enzymatic antioxidants ↑, phenolic and flavonoid compounds ↑.	(Jafari and Shahsavar, 2021)
	*Moringa olifera*	100 mM MT	Phenolic content ↑, lipid peroxidation ↓, and electrolyte leakage ↓, as well as photosynthetic pigment ↑.	(Sadak et al., 2020)
	*Solanum lycopersicum*	100 μM MT	Seedling growth ↑, proline content ↑, and total soluble sugar ↑.	(Altaf et al., 2022)
Salt	*Cucumis sativus*	100 μM MT	Photosynthetic parameters↑, ROS formation ↓, and antioxidant activity ↑.	(Wang L. et al., 2016)
	*Arachis hypogaea*	150 µM MT	SOD, CAT, POX, and APX activities ↑, phenolic content ↑, lipid peroxidation↓.	(ElSayed et al., 2020)
	*Ocimum basilicum*	1 µM MT	Phenolic and flavonoid content ↑, amount of rosmarinic acid ↑.	(Bahcesular et al., 2020)
Heavy metal	*Solanum lycopersicum*	100 µM MT	Anthocyanin, total phenolic, and flavonoid concentrations↑, and stimulate activities of PAL and CHS enzymes under Ni stress ↑.	(Jahan et al., 2020)
	*Cucumis sativus*	100 µM MT	PAL activity under control and stressful conditions ↑, and PPO, phenolic, and flavonoid activity ↑ under Fe^+3^ toxicity.	(Ahammed et al., 2020)
	*Oryza sativa*	50 µM MT	MDA and H_2_O_2_ content ↓, antioxidant activity ↑ under Cd stress.	(Jiang et al., 2022)

↑, Increase; ↓, Decrease.

## 9 Role of calcium in SM production under stress conditions

Calcium (Ca^2+^) is an essential element, present in plant shoots ranging from 0.1 to 5% on a plant dry weight basis (Thor, 2019). It is a vital component of plant cell walls, involved in cell signalling (nutrient and plant immunity signalling), and acts as a secondary messenger. The role of Ca²^+^ in maintaining membrane fluidity and integrity is crucial for preventing ion and metabolite leakage. It also fosters the accumulation of osmolytes (proline and glycine betaine), which assist in osmotic adjustment and protect cellular structures under stress conditions (Marques et al., 2016). Localization of Ca^2+^, primarily in the plasma membrane and other cellular compartments such as endoplasmic reticulum, golgi bodies, and plant vacuoles. In response to abiotic stress, protein kinase and phosphatase, both enzymes, mediate the coupling of Ca^2+^ and ROS signalling pathways using calmodulin-like protein (CAL) and CaM (calmodulin, a calcium-binding protein) as Ca^2+^ sensors (Zeng et al., 2015). Plant signalling processes are driven by the interaction of Ca²^+^ with its sensors. Primary Ca²^+^ sensors, such as calmodulin-like proteins (CMLs), calmodulin (CaM), calcineurin B-like proteins (CBLs), and calcium-dependent protein kinases (CDPKs/CPKs), are instrumental in mediating hormonal responses and stress signals (Zhu et al., 2022). Calcium chloride (CaCl_2_) applied topically proved more effective than calcium oxide and calcium chelate. For example, CaCl_2_ enhances PAL activity and up-regulates expressions of related genes such as *LcPAL1* and *LcPAL2*, thereby increasing the total phenolic content in *Cucumis melo*. It is reported that CaCl_2_ is advantageous for activating the phenylpropane pathway (You et al., 2024). Similarly, *Fagopyrum esculentum* was treated with 3% sucrose and 7.5 mM CaCl_2_ at the sprouting stage, and there was a high elevation in total phenolics and total flavonoid content. Dominant flavonoids occur in *Fagopyrum* sprouts, including C- glycosylflavonones (vitexin, orietin, isovitexin) and rutin (Sim et al., 2020). **Table 7** summarizes the role of Ca^2+^ and its donors in overcoming abiotic stress via the upregulation of SMs in different plant species.

**Table 7:** Exploring the role of calcium (Ca^2+^) in managing secondary metabolite production in diverse plant species under abiotic stress.

Abiotic stress	Experimental plants	Forms and concentration of Ca^2+^	Alteration in secondary metabolite production	References
Drought	*Triticum aestivum*	40 ppm Ca-P	Membrane stabilization↑, relative water content ↑, antioxidant formation ↑, and osmolyte content (proline, sugar) ↑.	(Mustafa et al., 2021)
	*Brassica napus*	75 ppm calcium oxide nanoparticle	Germination percentage ↑, photosynthetic pigments ↑, and improved seedling growth ↑.	(Mazhar et al., 2022)
	*Brassica napus*	100 mg L^-1^ Ca-NPs	Mineral absorption ↑, PSII and PSI efficiency ↑, gas exchange, and SM gene expressions ↑.	(Ayyaz et al., 2022)
	*Zea mays*	50 g L_–1_ CaCl_2_	Stimulation of stress-responsive genes ↑, grain production, and some phenolics such as gallic acid ↑.	(Abbas et al., 2021)
Salt	*Vicia faba*	16 mg L_–1_ CaP-NPs	Total soluble sugar ↑, proline content ↑, total phenolics↑, growth indices ↑, and oxidative markers (H_2_O_2_ and MDA) ↓.	(Nasrallah et al., 2022)
	*Sorghum bicolor*	6 g L^–1^ CaCl_2_	Relative water content ↑, osmotic balance in the mesocotyl and root ↑.	(Chen X. et al., 2022)
	*Gleditsia sinensis*	10 mmol L_–1_ CaCl_2_	Phenolic compounds, including L-phenylalanine and kaempferol ↑, affect ion homeostasis and ROS formation ↓.	(Guo et al., 2021)
Heavy metal	*Arabidopsis thaliana*	10 mmol L^–1^ Ca^2+^	Total phenolics, flavonoids, anthocyanin content ↑, and DNA damage ↓.	(Wang et al., 2014)

↑, Increase; ↓, Decrease.

### 9.1 Ca^2+^ under drought stress

Drought conditions are first perceived response through the root, resulting in the transmission of stress signals from the root to the aerial parts. Ca^2+^ promotes the synthesis of osmolytes, such as proline and glycine betaine, which help maintain osmotic balance and protect cells during drought. MYB, bZIP, WRKY, bHLH, CAMTAs, NAC, DREB, and MADS-box transcription factors, which are regulated by CaM, perform essential roles in maintaining normal physicochemical functions and enhancing stress resistance (Baek et al., 2023). In stress conditions, Ca^2+^ accumulation is high in the cytosol where it binds with CaM, thus regulating antioxidant activities and improving seed germination (Qin et al., 2019). Foliar-applied Ca^2+^ (50 mg/L) increased seed yield, sugar and starch content, and ionic balance in *Zea mays* under drought conditions (Abbas et al., 2021). Naeem et al. (2018) also reported in *Zea mays* that foliar spray of Ca^2+^ promotes the synthesis of osmolytes (proline and glycine betaine), which helps in maintaining osmotic balance and protects cells from drought. Furthermore, foliar treatment of CaCl_2_ in *Musa* sp. significantly raises the phenolics and flavonoids to overcome the toxicity of abiotic stress (Narwal et al., 2024). Applying CaCl_2_ in *Zoysia japonica* positively influences growth variables (seedling growth, chlorophyll, carotenoid content, and antioxidants) (Xu et al., 2013). In transgenic tobacco (*Nicotiana tabacum* L.) plants, the overexpression of StCaM2 (an isoform of CaM) increases their resistance to drought and salt conditions through the improvement in the functioning of PSII, which ultimately diminishes ROS formation and increases anti-oxidative enzyme activity in tobacco plants (Raina et al., 2021).

### 9.2 Ca^2+^ under salinity stress

Previous research demonstrated that salt stress stimulates Ca^2+^ accumulation, which acts as a bivalent cation for Na^+^ influx through a monovalent cation channel to maintain ion homeostasis. High accumulation of Na^+^ ions is the main toxicity in the soil. To achieve ion balance and control the outflow of excess Na^+^ ions, many transporters, ions, Ca^2+^ sensors, and their downstream interacting counterparts work in concert. Ca^2+^ is essential for enhancing salinity tolerance in plants through its role in signalling, root development, nutrient uptake, membrane stability, osmotic adjustment, and formation of antioxidants. Applying calcium phosphate nanoparticles (Cap-NP_S_) under saline conditions enhances the content of phenolic and flavonoid and antioxidant enzyme activities, and diminishes H_2_O_2_ accumulation (Nasrallah et al., 2022). Ca^2+^ concentrations (5, 15, 35 mM) significantly mitigate the saline toxicity in *Sorghum bicolor* via improved seedling growth, ion balancing, and antioxidant enzyme activity (Mulaudzi et al., 2020). Moreover, applying CaCl_2_ under salt stress increased the number of flower clusters per plant, several fruits per cluster, and the number of fruits per plant in *Olea europaea* L (El-Hady et al., 2020). CaCl_2_ elevated the level of various phenolic production, including L-phenylalanine, kaempferol, ferulic acid, and catechin, which responded negatively to the salinity stress in the case of *Gleditsia sinensis* Lam (Guo et al., 2021). These findings suggested that Ca^2+^ and its isoforms counteract the adverse effects of salinity via the synthesis and accumulation of some specific phenolic compounds in stressed plants. Moreover, overexpression of the CaM gene (*OsCam1-1*) under high salinity in rice significantly alters the expression of genes involved in cellular metabolism, hormone regulation, lipid and carbohydrate metabolism, secondary metabolism, and key cycles like glycolysis, tricarboxylic acid, glyoxylate, and signalling cascade (Yuenyong et al., 2018).

### 9.3 Ca^2+^ under heavy metal stress

For plants to respond to HM exposure, Ca^2+^ signalling is necessary for sensing, defence mechanism activation, oxidative stress control, and cellular homeostasis maintenance. Ca^2+^ alone or combined with other signalling molecules reduces metal uptake through the soil and minimizes ROS generation. For instance, foliage spray of CaCl_2_ enhances SOD, CAT, POX, and APX formation, stimulates NR activity, and enhances protein content in *Cucurbita pepo* under Ni stress (Valivand and Amooaghaie, 2021b). Similarly, CaCl_2_ was applied in the *Cicer arietinum* L. under Cd stress, and it invigorated the phenolic and flavonoid content to tolerate Cd toxicity by the formation of antioxidant molecules, encompassing both enzymatic and non-enzymatic categories (Ahmad et al., 2016). In another study, combined crosstalk of Ca^2+^ and MT synergistically increases the resistance in *Vicia faba* against As toxicity through regulation of the ascorbate glutathione cycle (Siddiqui et al., 2020). Role of calcium oxide nanoparticles (CaO NPs) attributed to its enhancement of Ca^2+^ absorption, photosynthetic pigments, ROS scavenging ability, reduction of As uptake, and translocation from roots to aerial organs in *Hordeum vulgare* L (Nazir et al., 2022). Similarly, Ca^2+^ not only decreases the uptake of Cd but also reduces Cd accumulation in plant cells by induction of the Ca^2+^ channel, enhances micronutrients like Na, P, K, and Mg through the soil via root absorption in *Fagopyrum esculentum* (Hakeem et al., 2022). These relevant findings underline the importance of exogenous Ca²^+^ in lowering metal accumulation, improving photosynthetic parameters, and higher the accumulation of SMs to achieve better yields.

## 10 Signalling molecules crosstalk for the regulation of the plant defence system

Signalling molecules (NO, H_2_S, MeJA, H_2_O_2_, ETH, MT, and Ca^2+^) respond in multiple interaction nodes, which conjointly upregulate prodigious metabolic activities in plants. In the context of prolonged NaCl stress, new insights emerge regarding the role of Ca^2+^ and H_2_S interactions in maintaining ion balance, regulating redox states, and influencing both primary and secondary metabolism (Khan et al., 2021). Plants use different sensors and signalling components against stress. One secondary messenger, cyclic ADP ribose (cADPR), triggers Ca^2+^ secretion from internal stores (endoplasmic reticulum) and operates effortless signalling in many plant and animal cells (Grams et al., 2024). Initial research in plants underscored the importance of cADPR in facilitating the action of NO on the activation of defence gene expression (Zhou X. et al., 2021; Naz et al., 2024). Cyclic ADP ribose activates NO synthase and catalyses NO production, upregulating CaM and Ca^2+^ signalling, influencing many other essential molecules in stress management. Calcium signalling activates many genes, including *CAMTA* (CaM binding transcription activator) and *MYB*, which implies a potential role during abiotic stress in *Aegilops tauschii* (Seifikalhor et al., 2019). Moreover, MT and other signalling molecules displayed positive interaction, which influences NO production through the activation of NOS-like enzymes in the arginine metabolic pathway (Aghdam et al., 2019; Hussain et al., 2024). Combinations of MT and NO produced NOMT (N-nitroso MT), which was recently discovered to play functions in the morpho-physiological activity of plants (Hussain et al., 2024). Melatonin and SNP-triggered NO significantly elevate isoflavone content by overexpression of cinnamic acid 4-hydroxylase (C4H) and PAL. It also amplified the gene expression of *PAL*, *C4H*, *IFS*, and *CHI1A*, which regulate isoflavone biosynthesis under abiotic stress (Yin et al., 2022). An experimental study (Sehar et al., 2023a) demonstrates that MT and MeJA synergistically influence S- assimilation, which upregulates ETH synthesis to dimmish the effects of heat stress. Ethylene regulates SOD, ascorbic acid activity and improves photosynthetic mechanisms in plants. Melatonin also contributes to preserving the amount of psbA and D1 protein in photosynthetic plants (Sehar et al., 2023b). Further, JAZs and MYC2, key regulators have been shown to play a crucial role in stress response by mediating JA signalling. Additionally, EIN3, its homolog EIL1, and the ERF-domain transcription factor ORA59 in *A. thaliana* demonstrate a positive interaction between JA and ETH signalling pathways under stress conditions (Zhu and Lee, 2015). Some studies revealed that NO and ETH are linked through mitogen-activated protein kinase (MAPK) signalling during stress (Wu et al., 2021). Mitogen-activated protein kinase positively regulates NO biosynthesis and NR activity in stress tolerance. It has also been reported that NO and MAPK markedly upscale the biosynthesis of H_2_S, which proves the positive correlation between NO and H_2_S (Bhuyan et al., 2020). However, the interplay between NO and H_2_S in PCD requires further exploration. While NO typically reduces oxidative stress, high levels of NO_3_^-^ may trigger ROS and MDA accumulation. H_2_S counters this via CsNMAPK signalling, but the MAPK inhibitor PD98059 weakens NO function and disrupts NO-H_2_S signalling, with underlying mechanisms still uncertain (Qi et al., 2019). Besides, the combined application of H_2_O_2_, CaCl_2,_ and SNP at the germination stage reversed the effects of saline toxicity in *Chenopodium quinoa*. Furthermore, their crosstalk improved α-amylase activity to promote seed germination, altering physiological mechanisms that help plants tolerate adverse conditions (Hajihashemi et al., 2020). It is well established that these vital signalling molecules regulate diverse aspects of plant development and positively react against stress conditions.

**Figure 3:**
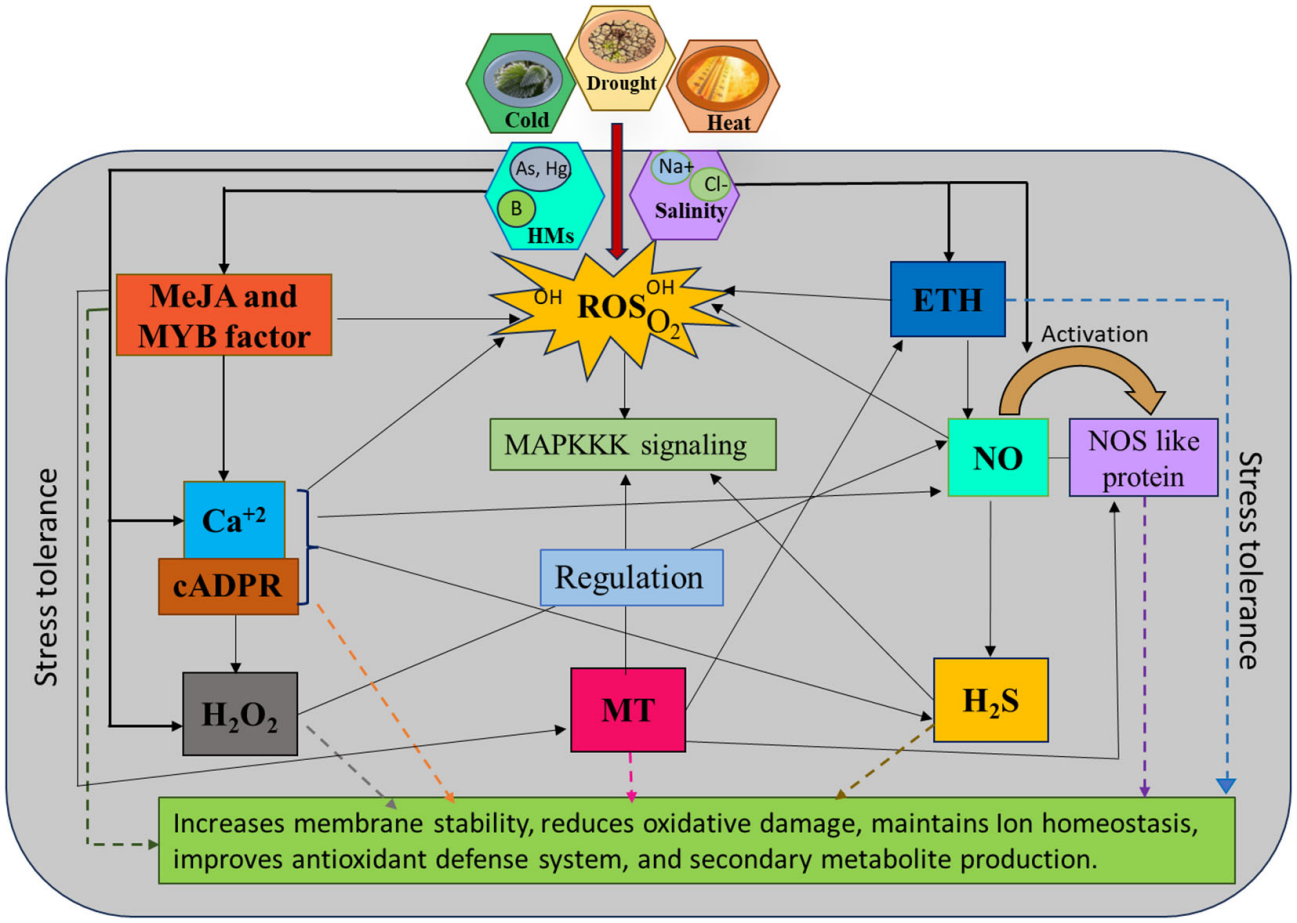
This intricate interplay between signalling molecules and their downstream effectors exemplifies the sophisticated regulatory systems plants have evolved to survive and thrive under abiotic stress. When plants face stress, they experience heightened levels of reactive oxygen species (ROS) accumulation, which can be alleviated by secondary metabolites activated by these signalling compounds. Arrows in the diagram indicate interactions between pathways, key donors and mediators involved in these processes including MYB transcription factors, sodium hydrosulfide (NaHS) or nitric oxide synthase (NOS) enzyme for H_2_S and NO production, sodium nitroprusside (SNP) as an exogenous NO donor, and cyclic ADP ribose (cADPR) for Ca²^+^ signalling which implement stress tolerance strategies through augmenting membrane stability, ion balance, and the generation of antioxidants (SOD, CAT, POX, and APX), and enhance metabolic reprogramming, ultimately contributing to plant resilience under harsh environmental conditions.

The above studies revealed that signalling molecules play significant roles in regulating SM production through their effects on enzyme activity, stress responses, signalling pathways, and oxidative cellular redox states. The crosstalk among diverse molecules, such as NO, H_2_S, MeJA, H_2_O_2_, ETH, MT, and Ca^2+,^ as stated in **Figure 3**, maintains a sophisticated signalling network that enables plants to respond to abiotic stresses effectively. Understanding their interactions could provide better ideas for enhancing stress tolerance in crops, ultimately improving agricultural resilience in challenging environmental conditions. The increasing intensity of numerous environmental stress factors makes plant survival much more challenging and crucial. We hope the signalling molecules will bring an entirely novel viewpoint to the field of study and encourage the scientific community to further research.

## 11 An innovative field in plant biology delving into the dynamic interplay between signalling pathways and secondary metabolite networks

### 11.1 Multi-omics approaches

Multi-omics approaches (genomics, transcriptomics, metabolomics, and proteomics) to map real-time interactions between signalling pathways.

Metabolomics provides direct insights into the status of metabolites and serves as a core point for connecting with other omics technologies to discover plant signalling molecules.

The interplay between plant metabolism and SMs is complex and multifaceted, and understanding these interactions is foremost for developing strategies to refine plant health and productivity in an altered environment.

### 11.2 Role of non-classical signalling molecules

Microbial VOCs (volatile organic compounds) are being found to modulate the production of secondary metabolites.

Bacterial volatile organic compounds (alcohols, aldehydes, alkenes, alkynes, benzenes, esters, heterocycles, ketones, sulphides, and terpenoids) induce defence and protect the plants from phytopathogens. The signalling pathways involving these are not well mapped.

### 11.3 Epigenetic regulation in response to stress

Epigenetic modification is a key tool for gene expression and SM production. Despite the advanced research, tools, and techniques in molecular biology and biotechnology, some questions regarding epigenetic modification remain unresolved. There are stress-induced epigenetic modifications (e.g., histone modification, DNA methylation) regulating signalling pathways and metabolite production, which remain unclear.

## 12 Conclusions and future perspectives

This review highlights the potentiality of signalling molecules (NO, H_2_S, MeJA, H_2_O_2_, ETH, MT, and Ca^2+^) in plant growth and development. These molecules regulate primary and secondary metabolic pathways to improve plant biochemical and physiological function and improve tolerance and resilience against abiotic stresses. It elucidates a better understanding of various signalling molecules that play putative roles in SM production by influencing plant responses specifically under stressful conditions. Under adverse conditions, signalling compounds typically rise, leading to the activation of SM-responsive genes and pathways that help to cope with stress. Subsequent research ought to focus on the intricate interplay among various compounds, such as NO, H_2_S, MeJA, H_2_O_2_, ETH, MT, and Ca^2^ **^+^** in regulating the synthesis of SM. Meanwhile, advancements in the medicinal plant field will come from using cutting-edge analytical techniques to identify novel SMs and their large-scale production. Biotechnological approaches, such as plant tissue culture techniques and CRISPR/Cas9 techniques, are useful for modulating and further identifying the genes involved in SMs synthesis. This could provide promising results and thereby overcome the issue of commercial exploitation of medicinal plants. Moreover, in changing climatic conditions where plants are being exposed to diverse stress situations, the inherent ability to produce SM can be increased by supplementing numerous signalling molecules. Such application would reduce the stress via SMs synthesis and help to achieve fundamental bioactive compounds that can be used in biopharmaceuticals. Further research is needed to deepen our understanding of the specific roles and mechanisms of various signalling molecules that offer the potential for developing stress-tolerant crop varieties with enhanced SM profiles.
